# 元田永孚の儒教思想の座標―儒教と皇道主義の間―

**DOI:** 10.12688/f1000research.51001.2

**Published:** 2021-05-07

**Authors:** 嚴錫仁 嚴

**Affiliations:** 1 Faculty of Humanities and Social Sciences, University of Tsukuba, Tsukuba, Ibakaki, Japan

**Keywords:** 元田永孚, 皇道主義的儒教, 革命論, 血統論, 皇道の訓解, Motoda Nagazane, Emperor centralist Confucianism, theory of revolution, theory of lineage, Lessons of the Emperor’s Way

## Abstract

本稿は、熊本実学派の一員として人生の後半期に明治天皇の側近となり、当時の西洋近代化の流れにあって、いわゆる保守反動の儒教主義的な政治・政策の復活を主導した、元田永孚の活動の思想的意義を論じたものである。革命論と血統論の検討においては、元田の主張を熊沢蕃山との比較、さらに東アジア儒学史に照らしてみることで、革命論を是認せず血統論を既定の事実として、君主の君徳培養に強調したのが元田の一貫した姿勢であり、それは日本思想史における典型的な儒学者の立場を示すものであったことを論じた。「皇道の訓解」の検討では、「三種の神器」の意味を儒教テキストによって裏付けようとする元田の言説を対象として、それは奥深くて知り難い皇道を教えやすい儒教によって補完するという意味を越えて、元田の思考では第一に儒教という絶対的・包括的なスタンダードがあり、その限りにおいて皇道の意義づけを行っていた、という見解を提示した。以上の検討を通して、元田は堯舜の王道政治を明治前期において本気で実現しようと臨んだ時代錯誤的な儒学者であり、また時勢に手遅れの認識もみられるが、それは彼が誰よりもまじめな儒学者であったことに起因するものであり、さらに彼の主張は後日の露骨な儒教的皇道主義とは違うものがあることを論じた。

## Abstract (en)

This paper discusses the ideological significance of the activities of Motoda Nagazane who, in the latter half of his life, became an attendant of Emperor Meiji as a member of the Kumamoto school of practical science. Whilst there were trends towards modernisation and Westernisation, Motoda Nagazane led a conservative reaction attempting to restore Confucianist politics/policies. I scrutinise the theories of revolution and lineage considering the history of East Asian Confucianism and comparing Motoda’s assertions to the views expressed by Kumazawa Banzan. In doing so, I assert that Motoda’s consistent attitude shows that he does not approve of the theory of revolution and that he regards the theory of lineage as an established fact. Thus, he highlights the cultivation of virtues in rulers, adopting the stance typically taken by Confucian scholars in the history of Japanese ideology. In ‘Lessons of the Emperor’s Way’, Motoda attempts to support the meaning of ‘The Three Sacred Treasures’ through Confucian texts. My evaluation of this text results in the view that in this discourse, Motoda transcends the significance of harmonising the deep and difficult ‘Lessons of the Emperor’s Way’ with Confucianism, which is easy to impart. I deduce that Confucianism was positioned above all else as the absolute/comprehensive standard in Motoda’s thought and that his endorsement of the Emperor’s way was proscribed within the ideological boundaries of Confucianism. Through the above analysis, I conclude that Motoda was an anachronistic Confucian scholar who truly endeavored to realise the kingship politics of Yao and Shun in the early Meiji era. While it is acknowledged that he was lagging behind his contemporaries, it is shown that this seemingly backward stance emanated from his serious Confucian scholarship. Further, his assertions differ from the plain-spoken Confucianist Emperor centralism that emerged in later years.

## 一　はじめに

本稿は、熊本実学派の一員として、人生の後半期に明治天皇の側近となり、当時の大勢の西洋近代化の流れにあって、いわゆる保守反動の儒教主義的な政治・政策の復活を主導した、元田永孚 （1818～1891） の活動の思想的意義を論じようとするものである。より具体的には、儒教思想を活動の基盤としながらも、天皇中心の皇道主義をそこに持ち込み、儒教経典を「皇道の訓解」 ^1^「皇道の註釈」 ^2^ として表現する元田の思想営為の内容如何を問い直してみたい。

沼田哲は彼の『元田永孚と明治国家』の冒頭において、元田を儒学者として規定してよいかという問いを立てて、元田自身の、横井小楠 （1809～1869） の実学を承ける儒者であるという言葉を引用しながら、「とりあえずこのように考えておいてよいであろう」と自答している ^3^。元田の儒学者像を改めて強調するような言葉でもあるが、微妙に引っかかる言い方でもある。なぜ、元田の思想を論じる誰もが認める当たり前のことを敢えて問いかけ、「とりあえず」という暫定的なニュアンスをも与える修飾語によって答えているのか。沼田にはこれ以上の説明がないので分からないが、本稿の方向に沿って勝手な想像が許されるならば、元田の思想営為には彼が模範とする小楠に比べて違うものがある、つまり儒教に加えた皇道主義的な言説の標榜がどこかで元田の儒学者像に微妙な影を落としている、ということも関係しているのではなかろうか ^4^。

ともあれ、元田の皇道主義的な言説は、しばしば彼の思想営為 （儒教思想）を否定的・消極的な方面に向かわせる要素として議論される。主に戦後の評価を指してのことであるが、たとえば、久木幸男は、元田の思想営為を明治儒教思想史の「きわめて特異」なものとして「元田的儒教」と呼び、それがために彼の儒教的な文教政策は「失敗」に終わったと厳しく批判する ^5^。その失敗の主因は、「元田が儒教とは全く異質の「天皇尊崇」を無理に組み入れ、「天皇尊崇」を中心に儒教道徳を再編しようとしたから」 ^6^というものである。あるいは、岩井忠熊は、元田を「明治の支配思想としての国家主義思想にもっとも関係の深い儒学者」であるといい、それは元田が天皇中心の政治としての「君権の拡充・絶対化」を目指していたこと、そしてその「君権の制限などということは、思いもおよばぬ」ような「元田の露骨な君主主義は、明治国家の立場において、すでに明白な限界をしめしていた」、と低く評価する ^7^。

もう一つ、これらとは方向を異にして、元田の儒教思想を肯定的に捉える松浦玲の立場も考えてみよう。朱子学゠実学゠政治学としての「儒教型理想主義」という概念をもって、明治 2 年 （1869） の小楠亡き後の「儒教型理想主義」の継承者として元田を位置付けるものであるが ^8^、全面的な肯定ではないのが注意を引く。「元田永孚のような、保守反動としての評価が確定している人物をここに持ち込むのは、物騒千万なのだが」 ^9^、「晩年の元田に対する是非論はしばらく措くとして」 ^10^というような条件付きの評価である。ここでの「保守反動」の意味が西洋近代化に対抗して、その弱点の是正を「孔子の学に求め」るという、儒教復活の主張 ^11^に限られるならば、そのレッテルは小楠にも張られるはずであるが、そうではないので、元田のみに適用される別の意味が含まれていよう。そして、それが「元田晩年の是非論」に関わるものとして、彼の晩年に色濃くなる「天祖を敬するの誠心が凝結してその根本をな」すという「国教論」 ^12^、さらには「皇室が我が国教育の中心でなければならない」という「国民教育論」 ^13^の提唱などを考慮に入れるならば、そのような批判めいた条件付きの評価の背後には、やはり儒教に加えた元田の皇道主義的言説が影響しているといってよいであろう。

以上、すこし強引な引用であったとも自覚しているが、元田の皇道主義的言説が彼の儒教思想への評価を否定的に、あるいは躊躇させる要因になっていることは確認できよう。だが、依然としてはっきりしない問題が残る。前述の引用を中心にしていえば、松浦は、元田が「最晩年」まで朱子学゠実学を堅持したというが ^14^、それならば、その儒教的立場と、評価保留の批判めいた「晩年の是非論」に潜められている皇道主義的立場との間隙はどう埋めればよいのか。

さらにそれは、元田の明治天皇の師傅としての役割においても相反する評価をもたらす。松浦のいう「儒教理想主義」は、具体的に朱子学的原則に従って「君主を聖人にする」ことを前提にするもので ^15^、つまり元田を小楠の「儒教理想主義」の継承者として評価する理由が、彼の「君徳培養」 ^16^としての朱子学的帝王学にあった。しかし前に引用した岩井は、元田のその帝王学の任務を「万機親裁」 ^17^として「君権の拡充・絶対化」を目指す「露骨な君主主義」といい、評価しない。元田の後半期活動の中心となる帝王学の比重を、松浦は儒教において、岩井は皇道主義において捉えているという、言い換えれば、元田の思想営為 （後半期）をめぐって、松浦的な儒教がベースとなる「皇道主義的儒教」なのか、逆に岩井的な皇道が中心となる「儒教的皇道主義」なのかが、衝突しているのである。

元田自身が「夙に尊王経世ノ志アリ」 ^18^ と述懐し、実際に儒教に加えた多くの皇道主義的言説が混在している状況において、どちらが優先的で真意であったかといった問いかけは、もはや無理・無意味なものかもしれない。にもかかわらず、こうした問いを立てるのは、少なくとも明治から昭和前期にかけての近代思想史において、元田の「皇道主義的儒教」 （あるいは「儒教的皇道主義」） が持つ象徴性が格別だったからである。その時代の思想史が、元田が希望していたものであったか ^19^、挫折・変質されたものであったか ^20^ は別として、近代儒教思想史に与えた元田の影響力が大きかったからである。

たとえば、昭和 9 年 （1934） 、「日本精神の作興」を求めて創立した「日本儒教宣揚会」が編纂した『日本之儒教』のなかに、当時の大東文化学院教授の藤沢親雄の演説文「明治天皇と元田永孚先生」が収録されている。そこでは、「儒教が我が国に移入せられまして以来次第に我が国固有の皇道即ち天皇道及び国体に醇化致しまして」というこの団体の常套語から始まり、「明治天皇の崇高なる世界観と人生観に対して異帯（原文ママ）なる精神的寄与をなしましたのは実に一世の大儒者元田永孚先生其の人であります」と、明維維新の功労者として「大儒者」の元田を持ち上げながら、彼の進講録の一つの「易」の講義を取り上げて、その原理の「王道精神」を「日清日露の戦」や「満州事変」「王道満州国の実現」として捉えている ^21^。

もう一つ、昭和 12 年 （1937） 、文部省より刊行した『国体の本義』の内容を敷衍解説するために、当時の学習院名誉教授の飯島忠夫に依頼して編纂した『日本の儒教』に載せられている元田に関する言及も、昭和思想界における彼の影響力を窺わせる一つの材料となる。冊子の最尾に置かれている「憲法と教育勅語と儒教」という章のなかのもので、元田を「朱子学者であつて、儒教を以て絶対の真理と信じて居た人である」と紹介し、元田が直間接的に関わった明治憲法と教育勅語について、「推古天皇以来幾多の波乱を重ねて議論されて来たところの日本の道は、この明治天皇が下し賜はつた憲法と教育勅語によつて、明快にして徹底せる解決に達し、従つて儒教の立場もまた明瞭となつた。憲法と教育勅語とは実に皇道の最高経典と謂ふべきである」と述べている ^22^。

元田の儒教に加えた皇道主義的言説は、国民道徳あるいは日本精神といった時代的イデオロギーの創出・普及に欠かせない重要な源泉の一つであった。そうした近代思想史の召喚に応じて、純粋でない保守反動的な儒学者元田のイメージも増幅していったのであろう。この「純粋でない儒教」は、さらにいえば、普遍的儒教と特殊・固有の儒教という問題にも換置できよう。儒教に加えた皇道主義的言説のゆえに「純粋でない儒教」という表現となるが、だからこそ日本の特殊・固有的な儒教ともなる、という意味である。本稿は、こうした問題意識を念頭に置いて、革命論と血統論への認識、「皇道の訓解」の意味という二つに焦点を当てて、元田の儒教に加えた皇道主義的言説の思想史的位置づけを再検討するものである。

## 二　革命論と血統論

前述の『日本の儒教』は、元田 （正確には教育勅語） によって「儒教の立場もまた明瞭となつた」といい、その証拠として「儒教思想がその中に混じている不純なる革命思想」を退けたことを挙げているが ^23^、実情はどういうものであったか、元田における儒教的な革命論と皇道主義的な血統論の関係について論じてみよう。

儒教の革命論は、『周易』革卦の「湯武、命を革めて、天に順ひ人に応ず」という語が示すように、民心を代弁する天命に委託して、有徳者による王朝交替を認める論理である。湯武の武力による「放伐」に加えて堯舜禹の間の平和的な「禅譲」を方法的な表象として、革命論は中国と韓国の歴史における王朝交替の正当性を担保してくれるものであった。こうした中国と韓国の歴史に対して、日本思想史では山鹿素行が『中朝事実』のなかで「 唯ひとり中国 （日本） は⋯⋯天神の皇統竟に違はず」 ^24^というように、王朝交替のない「皇統」 （天皇の血統） の持続性が強調されてきた。

ここで注意したいのは、革命論と血統論は、いずれかの一方に賛成を表明すれば、一方には反対しかない、いわゆる白黒思考のようなものであるかということである。論理的には二項対立的なものであっても、現実でははたしてどうであったか。本稿の目的に沿って先に方向を示せば、革命論 （とくに放伐論） に批判的だとして、それがただちに血統論の無条件的な支持にはならない、「中間的な立場」があるということになる。そしてその「中間的な立場」が、君主を有徳者に導こうとする儒教的な帝王学に関わっていることはすでに予測がつくことであろうが、急がずにまずは、二項対立の立場で血統論を支持する側の主張を拾ってみよう。

前述の素行が、中国と韓国の「革命」による王朝交替を「禽獣の相残ふ」 ^25^背徳的な行為として非難し、それがなく皇統が続いている日本こそ「中華文明の土」 ^26^だと誇っていた根拠は、「中国明かに三綱の 遺わするべからざることを知る。故に皇統一たび立ちて億万世これに 襲よつて変ぜず、⋯⋯三綱終に沈淪せず、徳化塗炭に陥らず」 ^27^という、儒教的スタンダードの「三綱」にあった。「徳化」を持ち出しているところも、素行の儒学者としての思想的位置を示すものといえよう。

こうした素行式の儒教的スタンダードによる革命論批判・血統論強調の主張は、神道家に至ると、より痛烈な展開を見せる。「禅譲の挙、倫理泯絶の禍を醞醸し、綱常淪斁の災を馴致す。⋯⋯革命の挙、天綱解紐の厄を 造いたし、地維脱結の変を揚ぐ。邪説の魁、暴行の首、孰か 焉これより大ならん」 ^28^と、堯舜の禅譲と湯武の革命 （放伐） を区別なく激しく罵る、垂加神道の流れをくむ松岡仲良 （1701～1783） の言葉である。王朝交替そのものを全面的に拒否するこうした姿勢の延長線上で、君主の資質などはどうでもよいという、無条件的な血統論支持の主張が登場してくるのは、ある意味当然の流れともいえよう。「只我国ハ 昏﹅ フ﹅ テ﹅ モ﹅ 天﹅ 君﹅ ハ﹅ 天﹅ 君、ト仰ギ戴キ奉リ、愚デモ宗領ヲ宗領トタツルヲ比莽呂岐ノ道トス」 ^29^ （傍点は筆者、以下同じ） と。

ところで、非難の語調や方向は異なるが、神 （天照皇） の子孫による統治という日本の特殊性を前提にして、無条件的な血統論を当然視する儒教側の主張もある。元田が自藩の先輩学者を除いて、ほとんど唯一その学問を是認していた熊沢蕃山 （1619～1691） の言説がそれである。蕃山は、湯武の徳が堯舜や文王に「及ばずして」、「放伐」という「徳に恥る」ことをやってしまったと ^30^、儒教の放伐論に冷ややかな態度を取りながら、日本の永続的な天皇統治の歴史を「必然の理」として、次のように擁護する。

天照皇は地生にをはしまさず。神武帝、其御子孫にして天統をつぎ給へり。⋯⋯然れども一度たゞ人となりぬれば、天統をつがず 地﹅ 生﹅にひとしきゆへに、天下をとりても帝王の号を得事不叶、三種の神器を身にそへ奉りて、天津ひつぎをふまん事は、天照皇の恐多く、且天威のゆるさぬ所あり。日本のあらんかぎりはかくのごとくなるべし。他の国はなき例なれ共、 日﹅ 本﹅ に﹅ て﹅ は﹅ 必﹅ 然﹅ の﹅ 理﹅也 ^31^。

天皇統治の当為性を、他国には例を見ない日本の固有の現象として打ちだす蕃山の主張は、彼の儒教受容の方法としての「時処位」論に基づくものであろうが、その天皇の資格を「たゞ人」の「地生」ではないところに求めていることは、やはり注意を引く。「天照皇」の血統は天皇統治を権威づける条件となるが、一方では「天統」「天威」のみにすがることによって、人君に必要な統治資質の涵養を疎かにし、または現実政治での役割を制限する論理にも繋がっていくからである。次の蕃山の言葉を見よう。

代をかさねて天下をたもつは天の廃する所なりといへり。しかれ共、王者は天神の御子孫にして地生にあらず。ことに日本にをいて広大の功徳をはします故、 天﹅ 下﹅ の﹅ 権﹅ 勢﹅ を﹅ ば﹅ さ﹅ り、給ひて、 や﹅ は﹅ ら﹅ か﹅ に﹅ し﹅ て﹅ 上﹅ に﹅ お﹅ は﹅ し﹅ ま﹅ せ﹅ ば﹅、いつまでも日本の主にてをはします道理にて侍り ^32^。

ここで蕃山は、血統論を問題視する『孟子』の論理を引き合いに、日本の「天神の御子孫」による世襲的統治を肯定している。だが、その天皇の「いつまでも日本の主にて」という永続的な地位は、「天下の権勢をばさり給ひて、やはらかにして上におはしま」すという、現実政治に関与しないことを条件としたものであった。徳川政権の下での自己検閲的な発言といえばそこまでであるが、蕃山には後醍醐天皇のような「王徳」のない天皇の政治参与を良しとしない言葉も見える ^33^。長い戦乱の末にやっと安定を取り戻した当時の秩序が、「天皇、道をしろしめさず、賢良を用ひ給はず、昔と時勢のかはりたる事をしり給はざりし」 ^34^というような存在によって、再び乱されることへの不安も、蕃山の天皇の政治参与を抑制する主張の一因であったに違いなかろう。

天下の人是を見て、威も力もなき人を日本の主筋とし、かくのごとくあがめ奉り主君となしてかしこまり給へるは誠に道ある君なり、我等いかで国・郡を給はりながら忠を存ぜざらむやと、むかし賊心ありし者も、たちまちひるがへして譜代の思ひをなせり。こゝを以世の太平すみやかなり。禁中をはしまさではいかで此徳あらんや ^35^。

「天下の人是を見て」の「是」はこの文のすぐ前において、戦国時代が収まり、武家の将軍と諸大名が参内して宮中の礼儀・音楽を見聞して感動するという光景をいう。「天下の人」が、武将たちの「威も力もなき人を日本の主筋」として崇敬する様子を見て、自分たちもそれを模倣することで「世の太平」が実現されるようになった、と蕃山はいう。要するに、「天皇の存在」そのものが、天皇の価値であり存在理由であるというものである。蕃山は、天皇に現実政治を主導していく役割を求めず、当然そこでは「王者」になるための積極的な帝王学も必要ないことになる。この点、元田とは大きな開きがある ^36^。

元田は、蕃山が経験していない天皇の「侍講」として、彼自身の表現で「未タ聖知発達ノ機ニ至ラス」 ^37^、「全体遅鈍の御天資に而、一と通り奉接候ては乍恐御不分りの様に奉伺候処」 ^38^、「或は狭急偏執の御失無きとも云難きを以て」 ^39^などといった、「地生」的な側面に対面していた。そこで、元田は「君徳培養」の教育を強調し、その教育は「人君」たる存在の必修要件であると、次のように進言する。明治天皇への初進講のときの言葉である。

抑人君天資ノ聡明学フニ頼ルニ非サルカ如シト雖トモ、苟シクモ智ヲ恃ンテ自ラ用ヰル時ハ其知ル所狭小ニシテ過不及ノ誤リアルヲ免レス。是ヲ以テ聖帝明王ハ必好ンテ聖人ヲ師トシ、其則リヲ取ル ^40^。

「人君」は血統的な資質に頼るのではなく、「聖人ヲ師トシ」て教育を受けなければならない。「聖人」は具体的に孔子、広くは儒教の教えをいう。そして、元田は、もし「人君」が教育を怠って「不徳」と見られる場合には、「環て視る者幾千万、其知識の日新、事業の月盛、我を蔑如する何を憚て為ざらんや」 ^41^と、人々から蔑視される事態を招くことになると諫める。さらには、「人君にして一念 愆あやまれば四海億兆の憂となり、一日怠れば千百年の患を 貽のこす」 ^42^といい、「人君」の教育は国家の「治乱興亡」に関わる大事な問題であると進言する。前述の松岡仲良の「昏フテモ天君」論はいうまでもなく、蕃山の「天統」「天威」に満足する血統論とも、異なる方向である。元田は単なる血統論の支持者ではないのである。

しかしとはいえ、元田をその反対の革命論の支持者として想定することも無理な話である。いうまでもなく、「大日本国は天孫一系の皇統万世に君臨す」 ^43^「皇統一系天壌窮り無し」 ^44^「神胤一系万古不易」 ^45^といった血統の永遠性をいう主張も、彼の思想活動の重要な一側面であったからである。もし彼のこのような言葉を、天皇の側近という地位による、あるいは歴史に再び呼び出された王政復古という時代的特殊性を反映する、たとえば徳富蘇峰が評した「高尚なる臨機応変者」 ^46^としての立場をあらわすものだとすれば、次の言葉には儒学者の一般論として革命論に反対する彼の立場が窺える。明治 16 年の新年進講の際に『論語』「道之以政」篇を講義するなかで持ち出した言葉である。

彼国テ堯舜禹ノ三代ト周公ノ成王ヲ輔ケテ天下ヲ治メマシタル世ノ三（原文ママ）、⋯⋯其余ハ多ク 天﹅ 下﹅ ヲ﹅ 奪﹅ ヒ﹅ 取﹅ リ﹅マシタル故ニ、所謂 利﹅ 己﹅ 主﹅ 義﹅ニテ、初メヨリ人君ノ徳ヲ以テ天下ヲ治メマスルノ主義ニテハコサリマセス。湯武ヲ始メマシテ斉桓・晋文・漢ノ高祖・唐ノ太宗・宋ノ太祖・明ノ太祖等孰レモ、己レ天下ヲ取リテ我功業ヲ為サント欲スルノ利己主義ヲ免レマセヌ故ニ⋯⋯ ^47^。

元田は、中国の王朝交替を「利己主義」によって「天下ヲ奪ヒ取」る行為だと指摘し、湯武の放伐をその利己主義の元祖的な行為として、蕃山よりも強い語調で批判していた。元田は革命論に反対しているのである。

このように論を進めてくると、元田は革命論と血統論をめぐって自己矛盾に陥っているかのように見えるが、しかし思想史における儒学者一般の見解はどうであったか、と問いを広げてみれば、別の視点も得られよう。儒学史において革命論に積極的な賛成を表した学者は、いったいどれほどであったかという問いである。

近代以前の学者のなかで革命論を支持した学者として有名なのは、明清の王朝交替期を経験し満州異民族の支配に絶望した黃宗羲 （1610～1695） くらいであろう。彼は『明夷待訪録』のなかで、「小儒は規規焉として、君臣の義を以て天地の間に逃るる所無しとす。桀・紂の暴に至りても、猶お湯・武当にこれを誅すべからずと謂いて、妄りに伯夷・叔斉の無稽の事を伝う。⋯⋯後世の君、父の如く天の如きの空名を以て、人の窺伺を禁ぜんと欲する者は、皆なその言を不便とし、孟子を廃して立てざるに至る。源を小儒に導くに非ずや」 ^48^、と革命論支持の論陣を張った。「小儒」たちの間違った世論操作によって、湯武の放伐よりも伯夷叔斉の荒唐無稽な故事が宣伝され、遂に孟子の革命論が廃止されるに至ったという論弁である。「天下の大害を為す者は君のみ」 ^49^という当時の君主制度を追っ払うためには孟子の革命論の導入が必要だという主張であるが、この言葉はかえって革命論が現実の思想界においては支持を得ていない寂しいものであったことを浮き彫りにしている。

孟子の革命論を公開的に攻撃した代表的な言説の一つに、北宋の名分論者の司馬光 （1019～1086） の主張がある。司馬光は、孟子が斉の宣王を相手に「君に大過有れば則ち諫む。これを反覆して聴かざれば則ち 位﹅ を﹅ 易﹅ う﹅」 ^50^と言ったことに対して、「孟子の言、以て驕君の非を格すに足らずして、ただ以て纂乱の 資もとを為すに足るのみなり」 ^51^と非難していた。

ところが、司馬光が指摘する「簒乱の資」は、実は孟子も心配していたことであった。「世を継いで天下を 有たもつ、天の廃する所は 必﹅ ず﹅桀・紂が若き者なり。故に益・伊尹・周公、天下を有たず」 ^52^と。すでに引用したように、蕃山はこの言葉を「代をかさねて天下をたもつは天の廃する所なりといへり」といい、孟子は王位の世襲そのものを否定しているかのように解釈しているが、この言葉の核心は「必ず桀紂」というところにあろう。孟子は、「天の廃する」放伐の対象に厳格な制限を掛けて、「簒乱」を禁止し、世襲も認めていたのである。さらに、「世衰え道微にして、邪說暴行 有また 作おこる。臣にしてその君を弑することこれ有り、子にしてその父を弑することこれ有り。孔子懼れて春秋を作る」 ^53^として、臣の忠的な論理を強調する『春秋』の大義名分論を宣揚したのも、孟子にほかならなかった。

そして、朱子は、孟子の革命論をあらわす、あの有名な「斉宣王問湯放桀」章の注釈において王勉の言葉を借りて、「ここの言は、 惟﹅ だ﹅下に在る者に湯武の仁有りて、上に在る者に桀紂の暴有る の﹅ み﹅は則ち可なり。然らざればこれ未だ簒弑の罪を免れざるなり」 ^54^と、放伐論に制約をかけることを忘れない。こうしてみると、孟子に代表される儒教の革命 （放伐）論は、君主を誡める意味が強く、実際の政権交替を扇動するものではなかったといえよう ^55^。言い換えれば、革命論は専制君主の悪政を防ぐための一種の警戒装置として、その実行を要求する儒学者は、少なくとも平時においては希少であり、大勢は血統による世襲を認めていたということである。

平時においては王位の世襲が当然のように受け入れられているなかで、儒学者たちが理想とする王道政治を目指して君主の有徳を確保しようとした制度が、まさに「経筵」であり「侍講」制度であった。朱子が 65 歳にして煥章閣代制兼侍講に任命されて即位したばかりの寧宗に『大学』を進講したことや、朝鮮の李退渓 （1501～1570） が 68 歳にして大提学・知経筵の任務で 16 歳に王位に就いた宣祖を相手として「戊辰六条疏」を上書し、「四箴」、「西銘」などを進講し、また「聖学十図」を献上したことは、その一例といえよう。元田が革命論を支持せず、かつ血統論に一定の距離を置いて「君徳培養」の教育を強調してやまなかったのは、それこそ儒教の伝統に倣って忠実に再現しようとしたことであった ^56^。

しかしそれにしても、今一つ釈然としない問題が残る。元田がそのような儒学者一般の立場を飛び越えて、「天孫一系」の日本的血統主義の主張を人一倍強く言い出していったのは、どのように理解すればいいのかという問題である。やはり皇道主義者の面貌によるものであるか。それとも、前述した徳富蘇峰の「臨機応変者」や小楠が批評する「利口者」 ^57^としての俊敏な状況認識が働いているものであろうか。要因は一つというより、濃淡の割合はともかくとして、幾つかの因子が複合的に絡んでいるのであろう。ならば、彼の儒学者としての立場により密着して、日本的血統主義の主張においてもそれは貫徹されているのか、もしそうだとすればその原理は何であったかを探してみることも、無意味ではないだろう。「華盛頓」論がその一端を窺う手がかりとなる。

アメリカの初代大統領のワシントンを、小楠が堯舜のように高く評価していたことはよく知られているところであるが ^58^、元田もその見解に同意し、むしろ小楠にはない、「華盛頓賛」「贅言」という専門的な一文を書くほどであった ^59^。「華盛頓賛」では、アメリカ独立戦争におけるワシントンの姿勢を、周の武王が殷の紂を討つ際に発したという『書経』泰西篇の内容に比肩し、さらにワシントンが大統領に就いて 8 年間務めた後に「位を辞し職を譲り恬然として旧閭に帰」ったことを、堯舜の「禅譲」と同様に捉えて、「其の聖徳大業豈堯舜と道を同じうする者に非ずや」と述べていた ^60^。「贅言」は、そのようにワシントンを称賛することは、西洋の「共和政治」を優越的なものに考えて、日本の「皇国の国体」を無視し貶めることではないか、という周囲からの批難に対して、それを弁解する内容のものである。

我が先皇の国を建つる、天孫の始めて茲の土に降臨したまひしより既に四海を以て一家と為したまひ、万民を視たまふこと赤子の如くその惻怛愛国の至、親ら其の労に任じて他人に託するに忍びたまはず、故に皇統一系万世に伝へて易りたまはず、其の恩沢寰宇に弥満し骨髄に浹洽す。⋯⋯彼の四年にて交代し民に常主無く自由に政を為すものと其の体裁固より同じからずと雖も、然れども其の天下人民の為にして一毫己が為にせざるの心は未だ曽て一ならずんばあらず ^61^。

「皇統一系万世に伝へて易りたまはず」と、日本的血統主義を擁護する姿勢は見てのとおりである。ここで注目したいのは、そうした「皇統一系」の「国体」は、「四海一家」「万民赤子」「天下人民の為にして一毫己が為にせざる」といった、いうまでもなく儒教の「王道政治」的な論理によって支えられてきた、という元田の言い分である。それは歴史的な事実というより、今侍講を務めている儒学者元田の責任意識・政治的目標が投影されているものと見るべきであると思うが、それはともかく、元田は続いて次のように天皇の世襲政治とワシントン的な共和政治の内実を、儒教の王道政治において一致させている。「嗚呼君臣の礼正しくして愛敬の道明らかに、同胞の義篤くして共和の実行はれ、法令簡明にして強制無く、民をして分に安んじ業を楽しみ各自主の域に至らしむるは是我が邦古来の国体なり。今果して能く是の如くんば則ち華盛頓も亦将に甘心せんとす。夫の君威を主とし束縛を専らにするが如きは則ち覇政の陋習我が皇国の体にあらざるなり」 ^62^と。「皇国の体」は「覇政」ではない「王道」を本質とし、その点においてワシントンも日本の血統主義に「甘心」するだろう、というのである。

「此一句ハ 舜﹅ 禹﹅ 授﹅ 禅﹅ノ要旨乃伝国ノ要言、我朝ノ三種ノ神器ノ意義ノ如シ」 ^63^とあるのは、元田が明治 18 年 （1885） の書経講義のなかで堯舜禹の間の「禅譲」の際に発せられたという「人心惟危～允執厥中」句を解説したときの言葉である。儒教革命論の一軸をなす「禅譲 （授禅）」を拒否するところか、日本的世襲論の象徴である「三種ノ神器」と同格の事柄として譬えている。前記した神道家の松岡仲良からすれば、到底あり得ないことになろう。といっても、これは元田が内心では革命論に加担していたなどではなく、革命論か血統論かの二項対立的な思考を乗り越えて、というより、彼の関心はそういう理屈的なところにはなく、堯舜禹と古代日本に共通して行われていたという理想社会を、この時代にもう一度実現したいという願望の表出として捉えるのが妥当であろう。共和制のワシントン賞賛もそうした脈略で理解すべきであろう。

だが、元田の「華盛頓ハ堯舜以来ノ聖人、或ハ優ル所アルモ知ルヘカラス」 ^64^といった評価は、出仕後の後半期に至ってはほとんど見られなくなる ^65^。しかし、ワシントン論に投影されている儒教の理想社会を実現すべく、その中心となる君主に対する教育の重要性を強調する姿は、晩年まで変わることがない。次の言葉は元田 70 歳のとき （明治 20 年） の新年進講で『周易』の乾卦を講義したもので、「君体」として血統的な世襲を前提し、「君徳」培養を力説している。

御国ノ御 君﹅ 体﹅ハ、全ク天道易理ニ御符合ノ御正体ニテコサリマスル、其君体ハ天ノ変リナキカ如ク一定不易ノ者ニテコサリマスレトモ、 君﹅ 徳﹅ニ於キマシテハ、乾々剛健ニシテ息ミマセス、須臾モ撓ミ怠リナク、朝廷百官天下万民ニ先立チテ、活溌運動天下ヲ率キ回ラス程ノ誠心カ、大土台ト相成リマシテ⋯⋯ ^66^。

「御国ノ御君体」つまり日本の皇統が間断なく継続しているのは、「天ノ変リナキカ如ク」当然のことであるが、それでも「君徳」は「須臾モ撓ミ怠リナク」錬磨しなければならない。さらに、元田の講義は次のように続く。

支那ヲ始メ外国ノ君体モ御国ト同ヤウニ万世不易ナル筈ニテコサリマスルニ、君体ノ動キナキニ泥ミマシテ、君徳モ怠リ滞リマシテ進ミマセヌヨリ、終ニ伏犠以後 革﹅ 命﹅ノ君トハナリ降リマシテコサリマスル。御国モ中世以後ハ恐レナカラ君体ノ動キナキニ御安心ニテ、君徳ノ剛健ヲ御欠アラセラレマシタル故、適々御 衰﹅ 微﹅ニモ相成リコサリマスルカ⋯⋯ ^67^。

中国の「君体」も日本と同じく「万世不易ナル筈」というのは、元田が王朝の世襲を日本だけの特殊事情ではなく、当たり前の一般論として考えていたことを伝えていよう。元田はそのうえで、中国では革命にあって王朝交替が繰り返され、日本では革命はなかったものの、王朝が「衰微」に陥った原因は、「君体」の不変性に安心して「君徳」培養に怠慢であったことによると指摘している。血統論を象徴する「君体」は既定の事実として、元田の主な関心はもっぱら「君徳」に注がれていたといえよう。だからこそ、「人君⋯⋯万一誠敬ノ心弛ミマシテ、一身ノ私欲ニ流レ怠リ荒ミマスレハ、天命忽チニ人君ノ身ヲ離レマシテ、天下ヲ保タレマスルコト相成リマセス」 ^68^という、より革命論の雰囲気を色濃く漂う、いわゆる「天命無常論」を明治天皇の前で進講しえたのであろう。

要するに、革命論を是認せず血統論を既定の事実として、なおかつ王道政治実現の主体たる時の君主の君徳培養に力を傾注しようとしたのが、この問題における元田の変わらない姿勢であった。それは東アジア思想史における、とくに日本においては蕃山をも乗り越えての ^69^、典型的な儒学者の立場を示すものであったといえよう。次に節を代えて、この元田の儒学者としての立場は、儒教経典を「皇道の訓解」などと位置付けるところでは、どのように表明されているのかを考えてみよう。

## 三　「皇道の訓解」

前で元田が儒教の「禅譲」を、「三種の神器」の伝達の意味と同じく捉える言葉を引用したが、神道 （皇道）の意味を儒教経典によって裏付けようとする具体的な取組は、この「三種の神器」をめぐる言及から確認することができる。次は、元田が侍講として出仕する前年の明治 3 年 （1870） に記したもので、ここでは儒教経典を「神教の注解」といっている。すこし長文になるが、煩をいとわず引用してみる。

①蓋上古の神聖聡明叡智の性、これに加るに寛容温厚発強剛毅の徳を以て天下に照臨し、教を万世に垂る。此時に当り文字の記するなく、書契の伝るなし、即ち地に対し日用に象なく、教を三種の神器に寓して、天下万世をして見て覚り易からしむ。蓋鏡の象は明なり、所謂聡明叡 智﹅の性なり。玉の象は 仁﹅なり、所謂寛容温厚の徳なり。剣の象は義なり 勇﹅なり、所謂発強剛毅の徳なり。②此三徳民生の始、之を天に受け性とする所のものは、 人﹅ 々﹅ 固﹅ 有﹅ せ﹅ ざ﹅ る﹅ こ﹅ と﹅ な﹅ し﹅。故に人君能敬して之を存し、推して以て天下に及せば、天下の人亦此三徳を明かにして、天下国家治平ならざることなし。③教約して旨遠し。実に言語文字の及ぶ処にあらず。神武天皇尊崇体認、崇神天皇欽承継述、 応﹅ 神﹅ 天﹅ 皇﹅に至り始て西土の経典を加えて 神﹅ 教﹅ の﹅ 注﹅ 解﹅となし、益斯道の精義奥旨を推演して、学路用功始て明かに、聖徳広運人に取て養を為すの誠意、実に堯舜と道を同ふす ^70^。

番号をつけて内容を三分したのは、今後の論議進行のために、いわば総論のつもりで設けたもので、それぞれこの問題に臨む元田の認識の方向が示されていると思ったからである。①では、「三種の神器」が象徴している徳目を、『中庸』の「智仁勇」の三逹徳に ま﹅ っ﹅ す﹅ ぐ﹅ に﹅配当している ^71^。最初の「蓋上古の神聖聡明叡智の性、これに加るに寛容温厚発強剛毅」という天照大神の徳を描写する語句は、『中庸章句』 31 章の言葉を模倣したものである ^72^。②では、神器の三徳を、天から人々に賦与されている「固有の性」、つまり『中庸』首章の朱子注釈に基づいて生まれながらにしてすべての人々に内在する人間の本性・本質として提示している。三徳の所有を天皇に限らず、衆人に広げて一般化・普遍化するという、ある意味大胆な解釈となるが、その拠り所もやはり朱子学である。そしてその観念において人君 （天皇）が先頭に立ち、三徳としての人間の本性を明らかにしていく、という統治原理が示されている。③では、「教約して旨遠し」の神器の真意が、応神天皇の『論語』の受容・伝達によって「神教の注解」となり、儒教と「道を同ふす」という、いわゆる神 （皇道）儒一致論が主張されている。一つずつ、もうすこし詳しく検討してみよう。

まず①での、元田が「三種の神器」の徳を『中庸』の「智仁勇」に配当して解釈する立場である。元田の解釈は、日本思想史の伝統を背景にしたものであるが、実はその伝統は元田の立場ほど明確ではない、というところに注意したい。元田による両者の結びつけを「まっすぐに」と表現したのは、それを念頭に置いたものである。元田が神器の意味をめぐって日本思想史のなかでとくに言及しているのは、北畠親房と熊沢蕃山である。

其後源親房神皇正統記ヲ著ハシ、三種神器ノ徳ヲ説キマスルニ、此中庸ヲ引証ニ致シテコサリマシ、熊沢了介ニモ三種神器ノ註解ハ中庸ニシクハナシト申シテコサリマスレハ ^73^、

ところが、親房の『神皇正統記』の解釈は、元田の言明とは違って実際には「中庸ヲ引証」していない。『神皇正統記』には、「鏡ハ一物ヲタクハヘズ。私ノ心ナクシテ、万象ヲテラスニ是非善悪ノスガタアラハレズト云コトナシ。其スガタニシタガヒテ感応スルヲ徳トス。コレ正直ノ本源ナリ。玉ハ柔和善順ヲ徳トス。慈悲ノ本源也。剣ハ剛利決断ヲ徳トス。知恵ノ本源也」 ^74^とあり、智仁勇ではなく、「正直」 （鏡） 、「慈悲」 （玉） 、「智恵」 （剣） を表象している。『中庸』の内容とは名目も異なり、徳目の配当もずれているのである ^75^。和辻哲郎が、「親房のこの解釈は極めて明瞭であつて、儒家の智仁勇三徳による解釈と混同される筈はないと思はれるのであるが、どういふ理由によつてか、少なからぬ学者がこの混同に陥つてゐる」 ^76^と指摘したとおりである。

もう一つ、元田は、蕃山の神器論も自説の典拠として引用している。蕃山の『大学或問』には、「智・仁・勇は天下の達徳なり。此三種の象を注解して経伝とせば、これに過たる神書あらじ。三種の注解は中庸にしくはなし」 ^77^という言葉がある。しかし蕃山の神器の解釈は、『中庸』のみの忠実な反映ではないのが、元田の立場と比較される。蕃山は『神道大義』のなかで「夫神道は正直を以て体とし、愛敬を以て心とし、無事を以て行とす」 ^78^と「神道」を定義しながら、それらの徳目は儒教においては「智仁勇の三にかよへり。正直は知なり、愛敬は仁なり、無事は勇なり」 ^79^と対応させる。また『三輪物語』では、「鏡は心の神明にして、虚霊不昧にかたどれり。天にありては日光とし、事におきては正直とす。玉は心の温潤にして、慈愛恭敬なるにかたどれり。天にありては月光とし、事におきては委曲とす。剣は心の剛強にして、堪忍裁断なるにかたどれり。天にありては星光とし、事におきては威武とす」 ^80^と解釈する。

こうした蕃山の解釈について、和辻哲郎は、「彼もまたこれを智仁勇の「象」と解するのではあるが、しかし正直、慈愛、断の三者をそこに結びつけざるを得なかつた」といい、「ここに太神宮の神託が影響してゐることは否定できないであろう」と指摘している ^81^。蕃山の解釈には依然として日本固有の神道的な立場が残っているという主張である。つまり、その蕃山の解釈との比較において、神器゠天照大神の徳を『中庸』の言葉に即してそのまま忠実に引用する元田の、儒教中心の解釈が浮き彫りになってくるのである。ちなみに、儒家神道を主張した山崎闇斎は、「鏡は分明なるを表し、玉は曲妙なる心を表し、剣は知恵の利を表し」 ^82^と、神道的な解釈を示していた。こうしてみれば、儒教理論を利用して神道を説明するという神儒一致の儒家神道は、明治の元田に至って「粹然たる」理論的な一致をみた、といっていいかもしれない ^83^。

次に②での、神器の徳目を人々の普遍的な性とし、それを統治原理に結び付ける見解を考えてみよう。まず普遍的な性に関して、すでにそれは朱子学の影響によるものといったが、具体的には、元田の「之を天に受け性とする所のものは、人々固有せざることなし」という言葉が、『中庸章句』 1 章の「天命之謂性～修道之謂敎」に対する、「蓋し人、己の性有るを知りて、其の 天﹅ に﹅ 出﹅ る﹅を知らず、⋯⋯聖人の教有るを知りて、吾の 固﹅ 有﹅する所の者に因りて、之を裁するを知らず」 ^84^という朱子の注釈に基づいていることによる。神器の徳目を人間の本性として把捉する元田の別の言葉を紹介すれば、「天祖ノ詔ハ乃此処ノ天命スルノコトニテ、三種ノ神器ハ乃此処ノ之ヲ性ト云コトニテコサリマスル」 ^85^、「故に上古の神聖三種の訓を示し、人々本性の徳を覚りて、之を全ふせんことを欲するの皇極とし、其これを学ぶの道は堯舜禹の欽明精一執中、孔子の克己復礼、大学の格物致知誠意正心修身、中庸の博学審問慎思明弁篤行、孟子の察識拡充一として本性に復るの法に非ざるはなし」 ^86^、などがある。

ところで、「天命の性」としての人間の本性は聖人と衆人との間に区別がない、という朱子学の議論は、孟子の性善説を引き継いだものである。朱子は、「聖人も我と類を同じくする者なり」という『孟子』の言葉について、「聖人も亦た人ならくのみ。其の性の善、同じからざる無きなり」という注釈を施した ^87^。だが、聖人と衆人が同じであるというのは、あくまでも善なる本性という抽象的・理論的な領域においてのことで、現実的な身分や階層の差異を無視する、いわば近代的な意味での人間平等論的な主張ではないことはいうまでもない。「聖人も人間であるにすぎない」という朱子の注釈には、聖人の特別な地位を奪うのではなく、誰でも聖人になりうるという衆人側の道徳的な奮発を催促する意図が込められていよう。朱子学では聖人に到達するための具体的な方法を「居敬」と「窮理」として定型化したが、元田のいう、これまた朱子学の重要な理論の一つである「復性論」に触れて「本性に復るの法」として列挙している堯舜禹から孟子までの工夫法が、それを反映していることは疑いない。神器が表象する徳目の所有を天皇に限定せず、人々の普遍的な性として位置付ける元田の解釈は、そうした朱子学の立論を駆使して立てられたものであった ^88^。

そして、元田はその朱子学的本性論を彼の構想する統治原理に適用していくが、この論法は明治 17 年 （1884）、伊藤博文に提出した「国教論」 ^89^という一文にも一貫してあらわれているので、それを引用して元田の立場を検討してみよう。

蓋し我の所謂 天﹅ 祖﹅ を﹅ 奉﹅ ず﹅ る﹅とは、かの釈迦を奉じ耶蘇を奉ずるその道固より異る。而して又祠官誦呪の如きに非ざるなり。天祖の徳は智仁勇にして人心の神府に賦在す。特に天祖の独得にあらずして伝へて列祖今上の神髄にあり。特に列祖今上の所有ならずして、 人﹅ 々﹅ の﹅ 固﹅ 有﹅ す﹅ る﹅ところなり。故に吾が霊智を磨き、吾が大仁を拡げ、吾が神勇を振ひ、以て身を修め、国を治め、天下を平かにするは、乃ち我が天祖を奉ずる所以なり ^90^。

「天祖の徳」゠智仁勇が天皇のみならず人々にも内在しているという主張が、ここにも繰り返されているのは見てのとおりである。元田の持論の一つといっていいだろうが、それはともかく、ここで注意したいのは、その主張が向かう帰着地である。元田は、「人々の固有するところ」となった「天祖の徳」を、今度は各自の努力で極大化して修身治国平天下に貢献する、それが「我が天祖を奉ずる所以」であると論を展開していく。つまり、朱子学の本性論を借りて三種の神器を説明する目的が、「天祖を奉ずる」として皇道を絶対化するところに収斂されていくのである。別の言葉を引けば、「今日ノ急務ハ天祖ノ詔ニ基ツキ神器ノ徳性智仁勇ノ三徳ヲ拡張致シマシ、忠孝ノ教ヲ修メマシテ」 ^91^というものである。

しかし、元田の「天祖を奉ずる」ということは、人々に求められる一方的な責務ではなく、ある先決条件の下で出されたものであることを見逃すべきではない。君主 （天皇）の先導と百官の補佐を、人々の責務に先立って要求しているということである。上引用に続く言葉の、「故に天子これを用ひ、以て明徳を天下に明らかにし、大臣百官これを用ひ、以て天子を輔けて国家を治む、士民これを用ひ、以てその身を修め、その所を安んず」 ^92^という、天子―大臣百官―士民の順番で、それぞれが担う違う役割を挙げていることがそれを示している。さらに、「国教論」の別バージョンでは、はっきりと天皇以下の政治指導者たちの「率先」を以下のように強調している。

其の天祖を奉ずるや、非礼拝祭祀虚文の謂に非ざるなり。即ち天祖の教を奉ずるなり。⋯⋯今天子と皇族大臣群僚百辟、一意にして之を奉じ、確乎として抜かず、以て 億﹅ 兆﹅ に﹅ 率﹅ 先﹅ す﹅ る﹅ときは、則ち億兆亦た将に感じて興起することを観る所有り ^93^。

「億兆」の責務に先立って「天子」の「率先」を要求するという観念は、すでにこの節の冒頭で引いた明治 3 年の「教育大義私議」のなかの「人君能敬して 之﹅ を﹅ 存﹅ し﹅、 推﹅ し﹅ て﹅以て天下に及せば」という言葉にも示されているが、それが『大学章句』経 1 章の「大学の道は、明徳を明らかにするに在り、民を 親あらたにするに在り」という、これまた朱子学的な統治原理に依拠していることも容易に推察することができる。

もう一つ引けば、『論語』為政の「道之以政」章の講義において、「徳ハ即チ人君躬ニ行ヒ心ニ得ル所ノ実徳、譬ヘハ人君躬親ラ孝弟ノ実ヲ尽シテ民ヲ孝弟ニ導キ、仁義ノ実ヲ尽シテ民ヲ仁義ニ導キ、躬ニ節倹ヲ行フテ民ヲ節倹ニ導キ、躬ニ天職ヲ尽シテ民ヲ職業ニ導クト云カ如ク、未タ人民ニ法令ヲ下スヲ待タスシテ、人君躬親ラ其行フヘキノ道ヲ尽スヲ此徳ヲ以テスト云コトナリ」 ^94^と、道徳の実践から日常の節約・仕事に至るまで人民に先立って「人君躬ニ行ヒ心ニ得ル」ことを要求する言葉からも確認することができる ^95^。

要するに、元田が「国教」として天皇を神聖視し皇道を絶対化するといっても、それは無条件的な追従ではなく、無制限の君権の拡張を助長するものでもなく、君主 （天皇）が「率先」して「躬ニ行」う、という儒教政治理論の伝統的教義をベースに置くものであった。こうした主張の延長線上で、③の「神教の注解」としての、儒教と「道を同ふす」という神儒一致論が出されてくるのであるが、しかしそこには全く一致とは言えない事情も見え隠れしている。次にそれを検討しよう。

元田が『論語』やそのほかの儒教テキストを「神教の注解」「皇道の註釈」あるいは「皇道の訓解」などとして位置付ける意味を、彼の言葉で探してみれば、「孔子ノ道ハ書ニ伝ヘ、我先王ノ道ハ神器ニ寓ス、書ニ伝フ故ニ講誦シテ教ヘ易ク、器ニ寓ス故ニ深奥ニシテ識リ難シ、唯道ハ一ナリ、故ニ書ヲ以テ器ニ参ヘテ其義覩ル可シ」 ^96^、「夫道は太神の訓に存す、人々自求めて足れり、然れども存誠致知の方、克己復礼の目、天下経綸の道、聖経に由らざれば其詳なることを得ず」 ^97^、などがある。神教・皇道と儒教の道は内実において同じであるが、神教の内容は奥深くて知り難いものなので、礼儀・道徳論から政治論に至るまで具体的な方法を具えて分かりやすく教えている儒教思想によって補うことをいうものである。しかもその儒教の受け入れは、選択的な補完ではなく、「書ヲ以テ器ニ参ヘテ其義覩ル可シ」「聖経に由らざれば其詳なることを得ず」というように必修的な補完として説かれている。いかにも儒学者としての元田の面貌を覗かせているものといえるが、それと関連して注意したいのが、応神天皇の儒教導入の宣揚と『論語』の特別な位置づけである。

唯此論語ノ書孔子ノ道ニ於テハ支那ノ書ニシテ 我﹅ 朝﹅ ノ﹅ 伝﹅ 書﹅ナリ。孔子ノ道ニシテ 我﹅ 日﹅ 本﹅ 人﹅ ノ﹅ 道﹅ナリ。⋯⋯扨又此書ハ辱クモ応神天皇ノ 御﹅ 伝﹅ 書﹅ナリト尊信スベキナリ。⋯⋯此論語ハ即チ天皇伝授ノ御書ト云テモ可ナリト存シ奉ルナリ ^98^。

『論語』は中国の書ではあるが、応神天皇の「伝授」によって「我朝ノ伝書」となったという。これに加えて、

この書は是れ応神帝伝授の書にして、皇道の訓解なり。何を以て之を云ふ、蓋し我朝にて道学を講ぜしは、帝より始まりて、我朝の書、此の書を以て 訓﹅ 謨﹅ の﹅ 権﹅ 輿﹅とす ^99^。

という言葉をみれば、元田において「皇道の訓解」と「我朝ノ伝書」は置換可能な概念であったといえる。重要なのは、元田が応神天皇による『論語』の「伝授」「伝書」を、文字や儒教の興起といった単なる先進文化の導入を超えて、「日本人ノ道」、または日本という国家の大計のはじまりをなす「訓謨の権輿」として、より日本的な特殊性を持たせながら位置付けている点である。この捉え方は、先輩学者たちの応神天皇の『論語』導入の意義を語る、たとえば、北畠親房の「此国ニ経史及文字ヲモチヰルコトハ、コレヨリハジマレリゾ」 ^100^、山崎闇斎の「是日本経学のはじめなり」 ^101^、山鹿素行の「ここに於て始めて書籍を伝へ、大いに儒風を闡き、文教の興ること誠にここに在り」 ^102^といった一般的な文化現象の興起を伝えることと比較すれば、その特別な位置づけが鮮明になる。それは言ってみれば、伊藤仁斎が『論語』を「最上至極宇宙第一の書」 ^103^として、「万世道学の規矩準則なり。⋯⋯万世に通じて変ぜず、四海に準じて違わず」 ^104^という普遍的真理の側面を評価したとすれば、元田は「日本人の道」として日本の特殊・固有の側面を『論語』に託してあらわしていたともいえよう。

そして、元田が固有の「日本人ノ道」として特に強調したのが「忠孝」であるが、この徳目の解釈においては本来の儒教との衝突が生じている。次は明治 11 年 （1878） の経筵での「論語弟子入則孝章」講義の一節である。

我が国は天地開闢より天祖の一君ましまして、臣民を統治し子々孫々万世窮りなし。故に天下の大道は君臣に始まりて、万づの道理皆 此﹅ の﹅ 君﹅ 臣﹅ に﹅ 包﹅ 含﹅ せ﹅ り﹅。⋯⋯君臣の忠義と父子の親愛とを 合﹅ 一﹅にしたる、世界無比の至道純理なれば、 堯﹅ 舜﹅ も﹅ 夢﹅ に﹅ も﹅ 見﹅ ず﹅、 孔﹅ 子﹅ も﹅ い﹅ ま﹅ だ﹅ 説﹅ き﹅ 出﹅ す﹅ こ﹅ と﹅ 能﹅ は﹅ ざ﹅ る﹅ 所﹅ な﹅ り﹅。此の君臣の大道、上下古今に貫通し、其の中に父子の親、夫婦の和、兄弟の序、朋友の信、其の余細大の道、悉く成り立つなり ^105^。

元田は、「忠」を君臣間の徳目として解しながら、日本では歴史的にその「忠」と父子関係の「孝」 （親愛となっているが、この講義の主題や脈絡からして孝にいい直して差し支えなかろう） との「合一」がなされてきたという。そして、その二つの徳目を「合一」的に捉えることは、堯舜や孔子もできなかった、日本のみに伝わる「世界無比」の特殊の観点であり、さらに君臣間の「忠」はあらゆる道徳を包括する一番上位の徳目である、と説いている。続く言葉では、「忠義を重んじ、三歳の童子も忠孝に死することを忘れず。我が国固有の道にて」 ^106^といい、忠孝の日本的な固有性を強調している。

元田は以上のことを『論語』の孝弟概念に即して示しているが、儒教思想史の脈絡と齟齬しているのはいうまでもない。元田は忠の徳目を君臣関係において見出しているが、その原義はむしろ、『論語』の「人の為に謀りて忠ならざるか」 ^107^などの自分の誠意を尽くすという対自徳目として理解するのが一般的である。また、忠と孝を合一的に捉え、さらに忠が孝よりも上位の概念であるということも、『孟子』のなかの、人を殺した父瞽瞍のために天下を棄てて孝を選択する舜を肯定する場面 ^108^を思い出せば、本来の儒教を離れた日本的な解釈というべきものになろう。武内義雄が水戸学を取り上げて、「日本は国家があたかも一大家族のような関係にあって、忠と孝とが一致する。この忠孝一致を力説したのが日本道徳の特徴であり、水戸学の精神である」 ^109^といったのは、日本儒教における忠孝の位相を示すものであるといえよう。

ところが、その経筵進講から 4 年後の明治 15 年 （1882） 作成された同じテーマ （弟子入則孝章）の講義草案の内容は、それとは別のニュアンスを伝えている。まさに水戸学を指して、忠のみを優先するその主義を批判したものである。

近世水戸烈公ノ一藩ヲ振起セシ時ニ専ラ尊皇攘夷ノ説ヲ主張シ、忠義節操ヲ以テ人ヲ励マシ、天下其風采ヲ仰キタル時ニ、吾先輩ナル横井小楠カ其学弊ヲ論シテ、専ラ忠義ヲ尚ンテ孝弟ニ本ツカス、其弊必ス気ニ馳セテ国ヲ誤ルコトアラント云テ、⋯⋯横井ノ見識モ実徳ニハ不足モアリテ、悉ク道ニ当リタルニテモ無ケレトモ、其孝弟ニ本ツカサルト云テ、必ス其弊アラント云タルハ、真ニ道ヲ見ルノ識眼ト云ハサルヲ得ス。後ニ水戸ノ君子小人党派相敵シテ遂ニ国ヲ害ヒタルニテ之ヲ鑑ミサルヘカラス ^110^。

元田は小楠とは違って水戸学へのシンパシーを残し続けたといわれるが ^111^、ここでは、小楠が水戸学を批判する「専ラ忠義ヲ尚ンテ孝弟ニ本ツカス」の観点を是認して引きながら、その主義が結局水戸藩の内紛を引き起こした原因になったことを、痛く指摘している。 4 年前の経筵進講での忠中心の立場とは打って変わって、孝をベースに置く考え方の表明である。忠を君臣間の道徳にしても、孝に基づいて忠へと、「夫れ孝は親に事うるに始まり、君に事うるに中し」 ^112^というような『孝経』的な本来の儒教に接近しようとするこのような姿勢は、明治 21 年 （1888） の『中庸』 20 章の新年進講で「五達道」の順番を説明することからも窺える。

父子有親已下ノ四ツノ道モ素ヨリ重キ道ニテ、特ニ父子ノ親ハ其人ノ生ヲ受ケマスルノ重キヨリ申シマスレハ、父子ノ道ヲ先ニ致シマスレトモ、天下ヲ治メマスルノ上ヨリ申シマスレハ、君臣ノ道立テ始メテ天地モ位シ万物モ育シマスルコトニテ⋯⋯ ^113^。

『中庸』の「五達道」では、君臣―父子という順番になっていて、『孟子』の「五倫」が父子を先頭に置くこととは違う。この言葉はその理由を述べたもので、君臣の忠を先に出しているのは、「天下ヲ治メマスルノ上ヨリ」という政治的な目的によるものだという。さらに、明治 15 年 （1982） に頒布された『幼学綱要』が、「孝行」を「人倫ノ最大義トス」として一番目に置き、「臣ノ忠節」を「子ノ孝行ニ並ベテ、人倫ノ最大義トス」として二番目に配置したのも ^114^、本来の儒教を考慮したものと把捉できよう。

要するに、元田は「皇道の訓解」゠「我朝ノ伝書」として、その中身を日本的な固有性が色濃い忠孝の徳目によって表象していたが、そうしたなかでも、儒教と「道を同ふす」、もう一つ別の言葉を引けば、「孔子の教、彛倫道徳を主として、天下国家を経綸し、我が邦の道と同じきを以て」 ^115^という彼の観念の大前提のもとで、儒教と日本の道 （皇道）を調和する姿勢を保持していたのである。それはあるいは、「皇道の訓解」といって、奥深くて知り難い皇道を教えやすい儒教によって補完するという意味を越えて、元田の思考ではまず第一に儒教という絶対的・包括的なスタンダードがあり、その限りにおいて皇道の意義づけを行っていた、といっていいかもしれない。

## 四　結び―時代錯誤の儒学者

以上、元田における革命論と血統論の認識、「皇道の訓解」の意味という二つのテーマを中心に、儒教に加えた皇道主義的言説の思想的座標を検討した。元田の思想営為は、普遍的な儒教に比重を置く皇道主義的儒教なのか、それとも日本の特殊性が重視される儒教的皇道主義とみるべきかという両面を掲げながら、結果的に、前者の儒教中心主義に沿って議論を進めてきたという感も否めない。実を言うと、元田の資料のいたるところで目に付く「堯舜」 （政治） を称賛し、その政治が目的となる王道政治・愛民政治を志向する言葉に接して、本稿の着想の段階からそういう方向を考えていたといっていい。

これまですでに堯舜に言及する元田の言説を取り上げてきたが、ここで改めて直接的に堯舜の政治を目指そうとする元田の言葉を探してみれば、

蓋堯舜ノ君臣ヲ以テ密ニ 当﹅ 世﹅ニ望ムナリ ^116^。 （明治 4 年）

今日御講書ノ始ニ此堯典ヲ講スルモ、畢竟堯舜ノ道ガ即 今﹅ 日﹅ 現﹅ 実﹅ノ御目的ト相成訳ニテ⋯⋯ 今﹅ 日﹅人君タルノ道ヲ尽サント思召サルヽ上ハ、堯舜ヲ御目的ニ遊サルヽヨリ外ハ是ナキコトナリ ^117^。 （明治 5 年）

陛下ハ張飛ヲ愛ス。張飛ノ声ハ万人ニ超ヘタリ。今元田ハ堯舜ヲ以テ陛下ニ望ミ奉ル ^118^。 （明治 5 年）

当﹅ 世﹅ニテモ法ヲ取ラセラレマスルニハ、此堯舜ヲ御目的ト遊サレマシテ然ルヘキコトト存上マスル ^119^。 （明治 12 年）

堯舜天下ヲ治ムルノ道ヲ知テ、之ヲ実際 今﹅ 日﹅己レニ得テ、之ヲ行フ ^120^。 （明治 16 年）

などがある。明治 4 年の宮中出仕に際して三条実美との面接時のやりとりの言葉から、明 16 年の「書経堯典講義」の言葉まで、ここに挙げたものだけでも、元田の堯舜政治にあこがれる一貫した態度を確認することができる。しかも、その態度は儒学者一般の単なる口癖的な復古調の願望ではなく、本気で「当世」「今日」における実現を目指すものであった。

「誠意正心ノ実心術ノ微ヨリ工夫ヲ下シ⋯⋯治国安民ノ道理利用厚生ノ本ヲ敦ク」 ^121^するという、小楠とともに熊本藩での講学時代に築き上げた「実学」の理念に基づく、堯舜を含む言葉で代えていえば「堯舜ノ内ヨリ外ニ及フノ教化」 ^122^となる道徳政治の具現を、明治前期の「当世」において真剣に取り組んでいたのである。

そして、元田が堯舜を借りて「当世」に実現しようとしていた具体的な政治が、人民を愛し優先する王道政治・愛民政治であった。

唯人君宰相心術ヲ正シ、品行ヲ修メ、⋯⋯万機ノ政ヲ為ス時ハ、法制ヲ設ルモ、禁令ヲ出スモ、刑罰ヲ施スモ、教化ヲ布クモ、皆 民﹅ ヲ﹅ 愛﹅ ス﹅ ル﹅ノ余リニ発シ、石室ヲ造ルモ、鉄道ヲ開クモ、一利ヲ興スモ、一害ヲ除クモ、総テ民ヲ先ニシテ己ヲ後ニシ、所謂人ニ忍ヒサルノ心ヲ以テ人ニ忍ヒサルノ政ヲ行フ ^123^。

蓋シ人君ノ大徳ハ仁ニ止マリテ、治道ハ人ヲ愛スルニ在ルノミ。若シ 人﹅ ヲ﹅ 愛﹅ ス﹅ ル﹅ ノ﹅ 心﹅至ラサル時ハ、事業大ナリト雖、国家富強ナリト雖トモ、天職ノ仁ニ悖レハ其余ハ観ルニ足ラス。今陸海軍ヲ皇張スルモ人ヲ愛スルカ為メナリ。憲法民法ヲ布クモ人ヲ愛スルニ因テナリ。教育勧業モ人ヲ愛スルカ為メナリ。鉄道電信モ人ヲ愛スルニ由テナリ。凡ソ国家ノ施ス所、一トシテ人ヲ愛スル事ニ非サルコトナシ。若シ人ヲ愛スルノ心ヨリ出テスシテ徒ニ事業ヲ盛ニシテ、欧州ノ文明ト競争セント欲スルカ如クナラハ、則是国家ニ長タルノ人ノ心ニ非スシテ、民心亦服スヘカラス ^124^。

これが、儒学者たる侍講として、元田の目指す政治の最終目的地ではなかったろうか。しかし、新国家の青写真がまだ何も決まっていなかった小楠の時代とは違って、西洋近代化が積極的に進められていた時代のなかで、「道徳ハ政事法律ノ本体、政事法律ハ道徳中ノ手足」 ^125^というような堯舜の道徳政治が地歩を広げる余地はなかった。明治 12 年 （1879） に元田が中心となって進めていた「君徳培養ヲ主トスル」 ^126^という「侍補制」が廃止に追い込まれたことは、その辺の事情を象徴的に伝えている。その意味で元田の堯舜宣揚と儒教中心主義は保守反動であり、それが目指す王道政治の理想的な意義はともかく、「時代錯誤」的なものでしかなかった。

元田の儒教思想における時代錯誤的な要素は、探そうとすればいくらでもあろう。君主 （天皇）と人民の関係を父母と子の間に比肩して、人民の君主への従属的関係として設定したことも、その一つである。

舜ノ天下人民ヲ治メマスルハ、丁度 父﹅ 母﹅ ノ﹅ 子﹅ ヲ﹅ 愛﹅ シ﹅ 育﹅ テ﹅ マ﹅ ス﹅ルヨウニコサリマス故ニ、子ノ望ミマスル所、子ノ言ハント欲シマスル所ニ、 舜﹅ 親﹅ カ﹅ ラ﹅ 先﹅ 立﹅ マ﹅ シ﹅ テ﹅、四方ノ門ヲ開キ、四方ノ耳目ヲ明ケマシテ、サア言ヘ、サア願ヘ、カヤウニ致シテ遣ハサフ、カウ致スカ宜シイト引起シ引起シ致シマスルコトテ、中々後世西洋ナドノヤウニ下カチニ人民ノ驕リマスルヤウノコトハ、聊之ナキコトテコサリマスル故ニ、先ニモ神武天皇ノ御心ト同シ心ト申上マスル如ク⋯⋯ ^127^。

先に引用した明治 12 年講義の前文で、当世において堯舜を倣う理由について述べたものである。西洋の「下カチニ人民ノ驕リ」のこととは対照的に、日本では最初から舜の政治のように、天皇と人民は親子関係としてあった （あるべき）という。また元田においてその関係は、「人君ヨリ人民ニハ始メヨリ 愛﹅ 育﹅スルト申シマスル天職ノ約信カ備リマシテ、人民ヨリ人君ニハ亦始メヨリ保護ヲ受ケルト申シマスル天分ノ願済ヲ得マシテ居マスル」 ^128^とも説かれていて、人君には人民を育てる、人民には人君の保護を受けるという役割ないしあり方が「天職」「天分」として与えられているという。

こうした主張にあっては人民の政治参与、つまり当時の自由民権運動が否定されるのは当然といえば当然のこととなろう。

堯舜ノ道ヲ以テ天下ヲ治メント志スハ人君ノ事ナリ。人臣トシテ其職ニ在ラスシテ、茲ニ志スハ寧ロ位ヲ出ツルノ思ヒニシテ、或ハ民権論者ノ人民参政ノ権アルト云ニマガフト云者モアランカ、是大ナル違ヒナリ。凡ソ此ノ如キ論、皆道理ヲ知ラサル故ニ、当世西洋ノ説ヲ誤認シテ大ナル弊害ヲ生セシナリ ^129^。

天下統治は人君の固有の仕事なので、人臣がそれを僭し、人民が政治に参与することは、西洋の悪影響による「大ナル違ヒ」であると非難している。そして続く言葉では、人々に「仁義五常の性」が具われている側面においては「上下貴賤隔テナキ所」だといいながら、「然トモ其事ニ於テハ君臣ノ分アリ、大臣小臣ノ別アリ、士農工商ノ等アリ、出処進退ノ異ナルアリ。故ニ臣民トシテ其位ニアレハ其政ニアツカリ、其位ニアラサレハ其政ニアヅカラス」 ^130^と、『論語』の「その位に在らざれば、その政を謀らず」 （泰伯） という正名論的な論法に依拠して、自説を正当化している。

さらに、元田がこの延長線上で「君権」を強調することも、時代錯誤的な儒学者の一側面となる。

憲法を建てゝ、其の自由を与へ給ひ、各種の法律を設けて不法を制し給ふも、総て君徳中の事にて、民の権利は皆君権にありて、君権は、 君﹅ 徳﹅ の﹅ 勢﹅ 力﹅ 範﹅ 囲﹅を云ふなり。⋯⋯万の法律、一つも君主の大権に帰せざるはなし。我臣民たる者、賜ふ所の権利を拝取して、誰か敢えて君上に向ひ、民権を唱ふ者あるべけんや ^131^。

憲法を含めてすべての法律は最終的に「君権」に帰属するものなので、人民はただ君権によって与えられた権利を「拝取」さえすればいいという。人民が自由に政治に参加する、今日的な「民主」という観念は徹底的に無視されているのである ^132^。

以上、作為的な制度論より自然的な心性・道徳論の重視、君主と人民の関係を家族関係になぞらえて服従的な性格を持たせたこと、君権の強調と民権の軽視といった、元田の儒教思想におけるいくつかの時代錯誤的な主張を取り上げたが、ここで注意したいのは、それは彼が儒教思想を誤解したり間違って理解したことによるものではなく、むしろ誰よりもまじめな儒学者・朱子学者であったことに起因する、ということである。儒教の政治思想には、元田がよく進講の主題とする『書経』が典拠の「民は惟れ邦の本なり」 （夏書） という「民本」はあるが、上述の「民主」概念は本来欠落されているからである。

そして、元田の時代錯誤的な主張は、彼が皇道主義者であったことによるものでもないことも指摘しておきたい。元田以後、いわゆる「家族制国家」のもとで、人民 （臣民）の天皇への忠誠の責務がやたらに肥大していった状況を想起すれば ^133^、上述の時代錯誤の引用で傍点をつけたところ―「愛育」「親カラ先立」「君徳」―だけをみても、元田は儒教の教えに従って治者側の道徳的な涵養・率先を絶えず要求していたからである。その点で元田の儒教思想は、後日の「本邦固有の皇道及国体に醇化したる儒教」 ^134^を標榜する 露﹅ 骨﹅な儒教的皇道主義とは違うものがあり、本稿は主としてそのことを検討したものである。

## データ利用可能性

本稿の研究結果の基礎となるデータは、すべて本論文中に示されており、追加のソースデータは必要とされていない。

## Footnotes

^1^ 元田竹彦・海後宗臣編『元田永孚文書』第二巻 （元田文書研究会、1969 年） 、『経筵論語進講録』、109 頁。以下、同書は巻数表示を漢数字によって示す。なお、引用文の旧字体は常用漢字に変え、適宜句読点を加えた。

^2^ 元田竹彦・海後宗臣編『元田永孚文書』第三巻 （元田文書研究会、1970 年） 、『論議講義』、231 頁。以下、同書は巻数表示を漢数字によって示す。この他に「神教の注解」 （海後宗臣『元田永孚』日本教育先哲叢書 19 、文教書院、1942 年、190 頁） という表現もある。

^3^ 沼田哲『元田永孚と明治国家―明治保守主義と儒教的理想主義』 （吉川弘文館、2005） 。序論の冒頭で「元田永孚という熊本藩出身の儒学者が居る」 （1 頁） といい、注においてこの問題を提起している （17 頁） 。

^4^ 沼田は、小楠と違う元田の思想場面を、水戸学 （国体論的考え方） への親近感を持続していたこと （同書、53～54 頁） 、小楠が「堯舜三代之道」を政道のモデルとして掲げていたに対して、元田は「我国上古の天祖神聖の道」への回帰を主張したことなどを挙げている （162 頁） 。純粋な儒学者という点からすれば、小楠より元田が遠く離れているということはいえよう。

^5^ 久木幸男「明治儒教と教育―1880 年代を中心に―」 （『横浜国立大学教育紀要』 28 、1988 年） 251～259 頁。その失敗の主因は「元田が儒教とは全く異質の「天皇尊崇」を無理に組み入れ、「天皇尊崇」を中心に儒教道徳を再編しようとした」ことにあるという （259 頁） 。

^6^ 同論文、25 頁。

^7^ 岩井忠熊『明治国家主義思想史研究』 （青木書店、1972 年） 59～66 頁。

^8^ 松浦玲「日本における儒教型理想主義の終焉 （一） 」 （『思想』 571 号、岩波書店、1972 年 1 月） 、「実学と儒教思想―日本における儒教型理想主義の終焉 （四） 」 （『思想』 630 号、岩波書店、1976 年 12 月） 。

^9^ 松浦、前掲「日本における儒教型理想主義の終焉 （一） 」、38 頁。

^10^ 松浦、前掲「実学と儒教思想」、106 頁。

^11^ 土屋忠雄『明治前期教育政策史の研究』 （講談社、1962 年） 360 頁。

^12^ 海後、前掲『元田永孚』、73 頁。

^13^ 同書、86 頁。

^14^ 松浦、前掲「実学と儒教国家」、107 頁。

^15^ 同論文、109 頁。「要は、君主を、為政者は聖人であるべきだという朱子学的原則の例外としてはならないところにある。君主こそが先頭に立たねばならない」という論法である。

^16^ 元田竹彦・海後宗臣編『元田永孚文書』第一巻 （元田文書研究会、1969 年） 、『還暦之記』、129 頁。以下、同書は巻数表示を漢数字によって示す。

^17^ 同書、158 頁。

^18^ 同書、126 頁。

^19^ 石田一良は、元田の「教育勅語」を「家族制国家の理念」として把捉し、明治 30・40 年代に至って『明治民法』と穂積八束らによって「本来元田が希望したとおり」になり、この人間型は第二次世界大戦の終了まで日本人の中核を構成したという （『日本思想史講座 6 近代の思想 1 』、雄山閣、1976 年、232 頁） 。

^20^ 小倉紀蔵は、元田の使命は道徳を具えた天皇が中心となる儒教的システムを作ることであり、「元田のこのような意図がいかに挫折・変質し、その挫折と変質がいかにその後の日本を規定したか」を分析することが、元田研究の意義であるという （『朱子学化する日本近代』、藤原書店、2012 年、258 頁） 。

^21^ 『日本之儒教』 （日本儒教宣揚会、1934 年） 204～217 頁。「異帯」は「偉大」の誤植か。

^22^ 飯島忠夫『日本の儒教』 （国体の本義解説叢書、教学局、1937 年） 99～100 頁。

^23^ 同書、102 頁。

^24^ 山鹿素行『中朝事実』、『山鹿素行全集思想篇』第十三巻 （岩波書店、1935 年） 42 頁。

^25^ 同前。

^26^ 同書、7 頁。

^27^ 同書、41 頁。

^28^ 松岡仲良『神道学則日本魂』、『近世神道論・前期国学』 （日本思想大系、岩波書店、1972 年） 253 頁。

^29^ 同書、260 頁。

^30^ 熊沢蕃山『集義和書』、『熊沢蕃山』 （日本思想大系、岩波書店、1971 年） 215 頁。

^31^ 同書、149 頁。

^32^ 同書、153 頁。『孟子』引用の「代をかさねて～天の廃する所なり」についてはまた後述する。

^33^ 蕃山は「帝王の天下」から武家政権に移った原因を、「謙徳を失ひ給ひし故に、天下の権威を失ひ給へりなり。⋯⋯其後王徳をとろへ、国々に我まゝなる者出来て⋯⋯」という （同書、149 頁） 。

^34^ 同書、11 頁。後醍醐天皇の余計な政治参与によって「一向武家の世とはなれり」というものである。

^35^ 同書、151 頁。

^36^ この問題をめぐる蕃山と元田の違いについては、森川輝紀「元田永孚と教学論」 （『埼玉大学紀要教育学部』 59 （1） 、2010 年、140～141 頁） から示唆を得た。

^37^ 元田・海後、前掲『元田永孚文書』一、『還暦之記』、129 頁。これは明治 4 年、初出仕の際の言葉。

^38^ 沼田哲・元田竹彦編『元田永孚関係文書』 （近代日本史資料選書 14 、山川出版社、1985 年） 148 頁。明治 11 年、下津休也に与えた手紙の一節。

^39^ 元田・海後、前掲『元田永孚文書』一、『当官日箚』、327 頁。明治 11 年の言葉。

^40^ 同書、『還暦之記』、128 頁。

^41^ 海後、前掲『元田永孚』、「君徳輔導の上言」、175 頁。

^42^ 同書、「教育大意私議」、193 頁。

^43^ 同書、「国憲大綱」、145 頁。

^44^ 同書、「教育大旨」、147 頁。

^45^ 同書、「君徳輔導の上言」、174 頁。

^46^ 徳富猪一郎『増補元田先生進講録』、「元田東野翁」 （明治書院、1910 年） 3 頁。山室信一はこの「臨機応変者」の言葉を挙げながら、元田の立場を「小楠の嗚乎血統論是豈天理順との堡塁を守るにはあまりに軽俊にすぎたし、自己のよって立つ地地盤の何たるかが見えすぎていた」という （「天皇の聖別化と国「教」論」、『近代熊本』 21 号、熊本近代史研究会、1981 年、3 頁） 。

^47^ 元田・海後、前掲『元田永孚文書』二、『新年講書始進講録』 （以下、新年進講録に略す） 62 頁。

^48^ 黃宗羲『明夷待訪錄』原君 （台湾中華書局印行、1969 年） 3 頁。

^49^ 同書、2 頁。

^50^ 『孟子』万章下 （徐徳明校点『四書章句集注』、『朱子全書』第陸冊、上海古籍出版社・安徽教育出版社、2002 年） 376 頁。

^51^ 司馬光『伝家集』巻 73 「疑孟」 （『影印文淵閣四庫全書』 1094 冊、台湾商務印書館、1986 年） 666 頁。

^52^ 『孟子』万章上、前掲『四書章句集注』、376 頁。

^53^ 『孟子』滕文公下、前掲『四書章句集注』、331 頁。

^54^ 『孟子集注』梁恵王下、前掲『四書章句集注』、269 頁。

^55^ 安炳周『儒教의 民本思想』 （成均館大学校出版部、1987 年） 54 頁。

^56^ ちなみに、「「沼山閑居雑詩」のなかの「嗚呼血統論是豈天理順」という一句によって、血統論に反対したといわれている小楠も、元田との談話の中では武王の放伐を認めていなかった。「文王の時既に三分の二を保ちたれば武王に到りて殷益小弱なる事知る可なり。此時に当りて時勢を云ふにも何ぞ殷を伐つに及ばんや。文王の如く只徳政を修め民を恤み、紂王は紂王にして存し置かば自ら衰へて終る可し。然るを我より事を起し人臣の道を失ふて数世連綿の国君を我手にかけて亡す事さてもさても道を得ざる事と伯夷は真誠明了に存じ込みしより馬を叩て諫たり」 （山崎正董『横井小楠遺稿』、『沼山閑話』、日新書院、1942 年、925 頁） 。一方で、元田は小楠の「嗚呼血統論」の詩に対して、「千百年来の活眼、天理の実、易道の本に達する者にあらざれば言ふ能はず」という、大いに肯定するような評価を与えていた （山崎正董『横井小楠伝記篇』、明治書院、1938 年、354 頁） 。要するに、この問題をめぐる彼らの見解も一貫しているとはいえない状況で、一方的な裁断は無意味であるということを伝えているのであろう。

^57^ 元田・海後、前掲『元田永孚文書』一、『還暦之記』、100 頁。小楠の「又曰吾兄利口ヲ止メヨ、利口誤ル所アラン。而シテ先生自ラ云、我 （゠元田） ハ利口者ナリ、人ノ及ハサル所」ということに対して、元田は「余自顧ルニ利口者タルコトヲ覚ヘス。⋯⋯先生ノ能ク余カ才質ヲ知ル所ナリト感服スルナリ」と受け止めていた。

^58^ たとえば、小楠は『国是三論』のなかでワシントンの業績を戦争終息、大統領の退任、共和政治などとして挙げながら、「殆三代の治教に符合するに至る」と評価していた （山崎、前掲『横井小楠遺稿』、40 頁） 。

^59^ この二文が著述された明確な時期は不明であるが、沼田哲の整理によれば、慶応元年 （1865） 以前の文として並べられている （「「元田永孚関係文書補遺」並びに「元田永孚文書目録」」、『青山史学』 10 号、1988 年、133 頁） 。なお、その全文は、原漢文を書き下し文にして前掲の山崎『横井小楠伝記』に収録されている。

^60^ 山崎、前掲『横井小楠伝記篇』、1142～1143 頁。

^61^ 同書、1130～1131 頁。

^62^ 同書、1131 頁。

^63^ 元田・海後、前掲『元田永孚文書』三、『書経講義』、74 頁。

^64^ 元田・海後、前掲『元田永孚文書』一、『還暦之記』、68 頁。元田が小楠の開国論とワシントン論を聞いて述べたもので、「安政乙卯ノ年」 （1855） の記録があり、元田 30 代前半の時期の言葉である。

^65^ 出仕後の言葉としては、明治 11 年 （1878） の「論語為政首章」の進講草案に「華盛頓の如きは、全く徳を以て国を興し、業を創めしなり」 （海後、前掲『元田永孚』、137 頁） というのがある。ただし、『元田永孚文書』二に収録されている『経筵論語進講録』の該当講義案には、この部分が省略されて見当たらない。

^66^ 元田・海後、前掲『元田永孚文書』二、『新年進講録』、85 頁。

^67^ 同書、86 頁。

^68^ 同書、71 頁。これは明治 18 年 （1885） 、元田 68 歳の『書経』虞書益稷の末節の新年進講の一節。

^69^ 天皇の現実政治への参与を否定し、君徳培養の帝王学の必要性も認めない蕃山の天皇論は、皮肉にも明治期において元田的な儒教主義に反対した福沢諭吉の言説に重ねられる。福沢は「帝室は万機を統るものなり、万機に当るものに非ず」 （『帝室論』、再版『福沢諭吉全集』第 5 巻、岩波書店、1970 年、263 頁） として、「帝室の神聖は政治社外の高処に止まりて広く人情の世界に臨み、其余徳を道理部内に及ぼして全国の空気を緩和せんこと」 （『尊王論』、前掲再版『福沢諭吉全集』第 6 巻、18 頁） を主張した。

^70^ 海後、前掲『元田永孚』、「教育大義私議」、189～190 頁。

^71^ 「智 （知） 仁勇」の四書での出典は、「子曰、知者不惑、仁者不憂、勇者不懼」 （『論語』子罕、前掲『四書章句集注』、147 頁） 、「知仁勇三者天下之達徳也」 （『中庸章句』 20 章、前掲『四書章句集注』、45 頁） 。

^72^ 「唯天下至聖、為能聰明睿知、足以有臨也、寬裕温柔、足以有容也、發強剛毅、足以有執也」。

^73^ 元田・海後、前掲『元田永孚文書』二、『新年進講録』、64 頁。

^74^ 北畠親房『神皇正統記・増鏡』 （日本古典文学大系 87 、岩波書店、1965 年） 60～61 頁。

^75^ 親房の智仁勇への言及は『東家秘伝』にみえ、「解曰、如レ玉ニシテ曲妙ナルハ、柔順ノ心ヲ表シ給也、仁也。如鏡にして分明ナルハ、正直ノ心ヲ表シ給也、心ノ本元也。如剣剛利ナルハ、決断心ヲ表シ給也、智也、勇也。尚書ニハ、剛・柔・正直ノ三徳トモ云、礼記ニハ、智仁勇ノ逹徳トモ云、其義皆一也」 （『神道大系論説編十八北畠親房 （上） 』、財団法人神道大系編纂会、1991 年、355 頁） とある。しかしここでも玉゠仁、鏡゠正直、剣゠智・勇として、元田のような配当とはずれが認められる。

^76^ 和辻哲郎『日本倫理思想史』下 （岩波書店、1952 年） 52 頁。

^77^ 熊沢蕃山『大学或問』、前掲『熊沢蕃山』、448 頁。

^78^ 熊沢蕃山『神道大義』、『蕃山全集』第五冊 （蕃山全集刊行会、1942 年） 13 頁。

^79^ 同書、14 頁。

^80^ 同書、239 頁。

^81^ 和辻、前掲『日本倫理思想史』下、447 頁。

^82^ 山崎闇斎『大和小学』、『山崎闇斎』上 （日本教育思想大系、日本図書センター、1979 年） 18 頁。

^83^ 「粹然たる」という形容は井上哲次郎の元田評価を借りたものである。井上は元田の儒教思想理解について、「決して仁斎、益軒、鳩巣等の下にあらず、⋯⋯が如きは粹然たる儒教の代表者といふを得べきなり」と評価していた （『日本朱子学派之哲学』、冨山房、1905 年、566 頁） 。江戸時代に鏡・玉・剣を儒教の智仁勇に対応させた儒者としては、林羅山「此三ノ内証ハ鏡ハ智也、玉ハ仁、剣ハ勇、智仁勇ノ徳ヲ一心ニタモツ義也」 （『神道伝授』、『近世神道論・前記国学』、日本思想大系、岩波書店、1972 年、12 頁） 、山鹿素行「玉は以て温仁の徳を表はすべく、鏡は以て致格の知を表はすべく、剣は以て決断の勇を表はすべし」 （『中朝事実』、前掲『山鹿素行全集思想篇』第十三巻、45 頁） などが挙げられる。

^84^ 『中庸』、前掲『四書章句集注』、59 頁、校勘記〔一〕。

^85^ 元田・海後、前掲『元田永孚文書』二、『新年進講録』、69 頁。

^86^ 海後、前掲『元田永孚』、「教育大義私議」、193～194 頁。

^87^ 『孟子集注』告子上、前掲『四書章句集注』、400 頁。

^88^ とはいえ、神器の徳目の所有を一般化する解釈は先輩の思想家にもあり、元田が引用する蕃山もそれに言及していた。「三種の象は孔門伝授の心法なり」 （『大学或問』下冊、前掲『熊沢蕃山』、449 頁） として、『中庸章句』の朱子注の言葉 （孔門伝授の心法） を引いて、「三種の神器は禁中におはしますといへども、其の象の心法は、上天子より下庶人に至まで、身を修るを以て本とするの義なり」 （『三輪物語』、前掲『蕃山全集』第五冊、13 頁） というものである。しかし、その理解は原論的な言及に止まり、元田の徹底した理解には及ばない。前述した蕃山と元田の儒教へのコミットの差異がここにも出ているといえよう。

^89^ 元田の「国教」という概念は、明治 12 年 （1879） に元田が起草し発表された「教育大旨」に対して、伊藤が「教育議」を奏上し、また元田が反論の形式で「教育附議」を著したことから顕在化した言葉である。元田が「祖宗ノ訓典ニ基ツキ」「祖訓ヲ敬承」することを「国教」とし、その中身を儒教に充当させることを提案したことに対して、伊藤はその儒教中心の教育方針を拒否したのであった （『教育勅語渙発関係資料集』第一巻、国民精神文化研究所、1938 年） 。

^90^ 海後、前掲『元田永孚』、「国教論」、204 頁。

^91^ 元田・海後、前掲『元田永孚文書』二、『新年進講録』、70 頁。

^92^ 海後、前掲『元田永孚』、「国教論」、206 頁。

^93^ 『教育勅語渙発関係資料集』第二巻 （国民精神文化研究所、1939 年） 297 頁。原漢文。海後宗臣よれば、明治 17 年に認められた「国教論」は 3 種あるというが （海後、前掲『元田永孚』、115 頁） 、この漢文体の文章には七月の記が付いている。伊藤に提出したものには 8 月の記がみえる。

^94^ 元田・海後、前掲『元田永孚文書』三、『論語講義』、341 頁。

^95^ さらには、「教育勅語」の末尾の「朕爾臣民ト俱ニ拳々服膺シテ」という句も、そこに示されている諸徳目を天皇も実践する義務があることを示している （苅部直『日本思想史の名著 30 』、ちくま新書、2018 年、198 頁） 。

^96^ 元田・海後、前掲『元田永孚文書』二、『経筵論語進講録』、121 頁。

^97^ 海後、前掲『元田永孚』、「為学之要」、173 頁。

^98^ 元田・海後、前掲『元田永孚文書』三、『論語講義』、231 頁。

^99^ 徳富、前掲『増補元田先生進講録』、「論語学而章」、1 頁。

^100^ 北畠親房、前掲『神皇正統記』、79 頁。

^101^ 山崎闇斎、前掲『大和小学』、4 頁。

^102^ 山鹿素行、前掲『中朝事実』、62 頁。

^103^ 伊藤仁斎『童子問』、『近世思想家文集』 （日本古典文学大系、岩波書店、1966 年） 58 頁。

^104^ 伊藤仁斎『論語古義』総論、『「論語」名家註釈書 （一） 』 （「論語」叢書、大空社、2011 年） 3 頁。

^105^ 元田・海後、前掲『元田永孚文書』二、『経筵論語進講録』、157 頁。

^106^ 同書、158 頁。

^107^ 『論語』学而、前掲『四書章句集注』、69 頁。

^108^ 『孟子』尽心上、前掲『四書章句集注』、437 頁。

^109^ 武内義雄「日本の儒教」、『武内義雄全集』 4 巻 （角川書店、1978 年） 114 頁。

^110^ 元田・海後、前掲『元田永孚文書』三、『論語講義』、280 頁。

^111^ 代表的に、沼田、前掲『元田永孚と明治国家』、53～54 頁。

^112^ 『孝経』開宗明義章 （『諸子集成補編一』、四川人民出版社、1997 年） 4 頁。

^113^ 元田・海後、前掲『元田永孚文書』二、『新年進講録』、90 頁。

^114^ 前掲『教育勅語渙発関係資料集』第一巻、33 ・ 44 頁。

^115^ 元田・海後、前掲『元田永孚文書』二、『経筵論語進講録』、158 頁。

^116^ 元田・海後、前掲『元田永孚文書』一、『還暦之記』、127 頁。

^117^ 元田・海後、前掲『元田永孚文書』二、『新年進講録』、3 ・ 5 頁。

^118^ 元田・海後、前掲『元田永孚文書』一、『還暦之記』、135 頁。

^119^ 元田・海後、前掲『元田永孚文書』二、『新年進講録』、39 頁。

^120^ 元田・海後、前掲『元田永孚文書』三、『書経講義』、4 頁。

^121^ 元田・海後、前掲『元田永孚文書』一、『還暦之記』、27 頁。

^122^ 元田・海後、前掲『元田永孚文書』二、『新年進講録』、43 頁。

^123^ 同書、13 頁。

^124^ 元田・海後、前掲『元田永孚文書』二、『経筵論語進講録』、143 頁。

^125^ 元田・海後、前掲『元田永孚文書』二、『新年進講録』、92 頁。

^126^ 元田・海後、前掲『元田永孚文書』一、『古稀之記』、179 頁。

^127^ 元田・海後、前掲『元田永孚文書』二、『新年進講録』、38～39 頁。

^128^ 同書、29 頁。

^129^ 元田・海後、前掲『元田永孚文書』三、『書経講義』、4 頁。

^130^ 同上。

^131^ 海後、前掲『元田永孚』、「論語為政首章」、139 頁。

^132^ 苅部直は、元田の「自由」「権利」の概念を検討し、彼の「道徳社会」では「個々人の「不羈自由」は完全に封殺される」と指摘している （『歴史という皮膚』、岩波書店、2011 年、224 頁） 。

^133^ たとえば『国体の本義』 （文部省、1937 年） に、「抑々我が国は皇室を宗家とし奉り、天皇を古今に亙る中心と仰ぐ君民一体の一大家族国家である」 （38） として、それゆえに「即ち我等は、生まれながらにして天皇に奉仕し、皇国の道を行ずるものであって、我等臣民のかかる本質を有することは、全く自然に出づるのである」 （32） とある。

^134^ 前掲『日本之儒教』、例言。

