# Supplementary material for: Motoda Nagazane’s standards of Confucianism – between Confucianism and Emperor Centralism
Source: F1000Res. 2021 May 7;10:272. [Article in Japanese] Originally published 2021 Apr 6. [Version 2] doi: 10.12688/f1000research.51001.2 (PMC8095323; doi:10.12688/f1000research.51001.2)
Supplement: Peer review history

# Peer review: Reviewer response for version 1

**DOI:** 10.5256/f1000research.54105.r83014

**Published:** 2021-04-22

**Authors:** Ryu Kataoka, Tomoya Masuda

**Affiliations:** 1 Graduate School / Faculty of Arts and Letters, Tohoku University, Miyagi, Japan

**○総評**

 　本論文は従来、皇道主義と儒教思想との間で揺れのあった、元田永孚の思想的評価を、元田の皇道主義的言説を分析することによって、「普遍的な儒教に比重を置く皇道主義的儒教」として確定したものである。その分析は優に学術的水準を満たしており、結論も妥当である。

 　強いて言えば、皇道主義と儒教思想かという二項対立的な問題設定の枠組み自体を突破しうるだけの鋭い分析がなされながらも、結論が結局は枠組みの中での評価の精緻化にとどまっている点が、惜しまれる。思想史的評価に際しては、言説分析にとどまらず、元田の社会的実践との関係も考慮に入れる必要があるのではないか。

 　以下、細にわたるが、確認を希望する点を列挙しておく。

**○誤字脱字の確認を要する箇所**

 　・注21の引用文

 　　「異帯なる」→「偉大なる」？

 　・注47の引用文

 　　「世ノ三」→「世ノミ」？

 　・注48の引用文他複数個所

 　　「傑」→「桀」？

 　・注131の引用文

 　　「あるべんや」→「あるべけんや」

 　・九頁四段落

 　　「加担していたなと」→「加担していたなど」？

**○日本語（訓読含む）表現としての妥当性の検討を要する箇所**

 　・三頁四段落

 　　「継ぎ手」→「継承者」？

 　・注18直後

 　　「懐述」→「述懐」？

 　・注48の引用文

 　　「源を小儒に導くに非らずや」→「源を小儒に導かるるに非ずや」？

**○文意が曖昧なため、表現の修正の検討を要する箇所**

 　・三頁二段落

 　「すこし大げさに聞こえるかもしれない理由によるものである。」

 　→(1)何の理由か、(2)誰に「大げさに聞こえる」のかが、一読して分かるような表現の工夫

 　※総じて三頁二段落の全体が、導入として迂遠に感じる。

**○内容に関する確認を要する箇所**

 　・「純粋でない儒教」（五頁一段落）、「まじめな儒学者」（一七頁最終段落）という用語はなにをもって「純粋」「まじめ」とするのか、論者の価値観が混入していることにならないか？

 　・五頁三段落

 　「革命論は中国と韓国の歴史における王朝交替の正当性を担保」

 　　→韓国の場合、そう言い切れるか？

 　・十四頁～十五頁にかけての段落

 　　→忠孝一致を単純に日本的と断定できるか？

 　たとえば『礼記』祭統に「忠臣以事其君、孝子以事其親、其本一也」。

 　もちろん、『論語集注』（八佾「君使臣以礼、臣事君以忠」）に「尹氏曰、 君臣以義合者也。故君使臣以礼、則臣事君以忠」、『朱子語類』巻十三に「父子兄弟夫婦、皆是天理自然、人皆莫不自知愛敬。 君臣雖亦是天理、然是義合。世之人便自易得苟且。故須於此説『忠』、卻是就不足処説」とあるように、「天理」である孝と「義合」である忠との区別を意識した上で、だろうが。

（該当する場合は要回答）統計分析および解釈は適切ですか。

対象外（統計を使っていない）

本研究は明確かつ正確に提示されたものであり、最新の文献を引用していますか。

はい

方法と分析について第三者による再現が可能となるよう十分な詳細が提示されていますか。

はい

結果の基礎となるソースデータはすべて入手可能で再現性を十全に保証していますか。

はい

結論は結果により妥当な裏付けを得ていますか。

はい

研究設計は適切で学術的価値がありますか。

はい

Reviewer Expertise:

片岡龍（東アジア思想史）増田友哉（近世日本国学、皇国主義思想）

We confirm that we have read this submission and believe that we have an appropriate level of expertise to confirm that it is of an acceptable scientific standard.

# Response

**Published:** 2021-04-23

**Authors:** Seogin Eom

**Affiliations:** University of Tsukuba, Japan

新学期のご多忙の時期に、ご丁寧に読んで下さり、適切なご指摘・ご教示いただきまして、心より感謝申し上げます。元田の思想史的評価においては彼の社会的実践の側面も考慮に入れるべきだというご指摘には、筆者も痛く共感しているところです。今回はそこまで論ずる余裕はなかったのですが、別の機会に譲りたいと思います。改訂版にてご指摘のあった曖昧な文章や脱字誤字等を訂正しました。

 内容確認の部分については、簡単ながら補足説明いたします。

 ・「純粋でない儒教」については、「純粋」か「不純」かのような価値的な区別ではなく、儒学者として儒教的言説のほかに、それとは別の性質の言説もまじえて表現したという意味で用いたものです。あえていうならば、壇君神話のようなものを儒教言説と一緒に提出する、といった習合的な傾向はほとんどみられない韓国儒教との比較的観点はあったかと思います。

 ・「まじめな儒学者」については、強調的表現です。

 ・「革命論は中国と韓国の歴史における王朝交替の正当性を担保。→韓国の場合、そう言い切れるか？」については、新羅最後の敬順王が高麗の王建に自ら帰順し、高麗と朝鮮の王朝交替のときに、「禅譲」の形式を取っていたことを、念頭に置いたものです。

 ・「忠孝一致を単純に日本的と断定できるか？」についてはご教示のような言説（『論語集注』『朱子語類』）にもよりますが、元田自身がそれを「世界無比」として持ち出していたことを踏まえました。

# Peer review: Reviewer response for version 1

**DOI:** 10.5256/f1000research.54105.r83015

**Published:** 2021-04-13

**Authors:** Ryoko Shimokawa

**Affiliations:** 1 Department of Japanese Culture, Faculty of Letters, Aichi Gakuin University, Aichi, Japan

本論文は、明治天皇の侍講となり、『教育勅語』の起草に関わるなど、明治の言論界で活躍した元田永孚の皇道主義的言説を分析し、その儒教思想の構造を明らかにしたものである。彼は、革命論を是認せず、血統論を既定の事実として王道政治実現の主体である君主の徳の涵養をめざした儒者の標準的思考の持ち主だった。さらに皇道（神道）の意味を、朱子学の文献、例えば『中庸章句』の三達徳の概念を用い論理づけようとした。北畠親房や熊沢蕃山がもっていた曖昧さを排除し、より儒教的解釈に徹したのである。そして、三種神器の徳目を天皇に限定せず、人々の普遍的性とする朱子学的本性論を展開した 。各自の努力でその徳を極大化して修身治国平天下をめざすという主張は、朱子の『大学』解釈を踏まえたものである。

 元田の思想は、国教として天皇を神聖視し皇道を絶対化したものだと言われるが、実際には儒教の政治論の伝統的教義にのっとっているのである。忠孝の徳について、日本では孝よりも忠を重視し、それが天皇中心思想につながると言われてきたが、元田永孚は、忠を強調した水戸学を批判し、孝経的な本来の儒教に近づけようとした。本論文は、結論として、彼がめざしたものを、堯舜の治政の再来、人民を愛し優先する儒教的王道政治と指摘する。幕末から明治の皇道的な思想の系譜の中で語られる元田の論理を、東アジア全体の儒教思想史の中に位置づけた本論文の結論は十分に説得力がある。

 ただ、元田の儒教思想を評価する際に、朱子学的論理と西洋的自然法思想との本質的類似性に着目し、天賦人権論の文脈に儒教を援用した中江兆民との違いにも言及があれば、明治期における儒教思想の果たした役割がより明確になったと思われる。けれども、元田の儒教思想の構造を明らかにした本論文は日本思想史研究、東アジア儒教史研究に大きく寄与するものと考えられる。

（該当する場合は要回答）統計分析および解釈は適切ですか。

対象外（統計を使っていない）

本研究は明確かつ正確に提示されたものであり、最新の文献を引用していますか。

はい

方法と分析について第三者による再現が可能となるよう十分な詳細が提示されていますか。

はい

結果の基礎となるソースデータはすべて入手可能で再現性を十全に保証していますか。

はい

結論は結果により妥当な裏付けを得ていますか。

はい

研究設計は適切で学術的価値がありますか。

はい

Reviewer Expertise:

日本思想史

I confirm that I have read this submission and believe that I have an appropriate level of expertise to confirm that it is of an acceptable scientific standard.

# Response

**Published:** 2021-04-23

**Authors:** Seogin Eom

**Affiliations:** University of Tsukuba, Japan

新学期の慌ただしい時期に、詳しく読んで下さり、また評価していただき、真にありがとうございました。

 ご指摘いただいた中江兆民との比較は、二人の民権をめぐる議論も含めて、別のテーマとしても興味深いものがあるかと思います。機会があれば挑戦してみたいと思います。
